# Experimental Research on Mechanical and Shrinkage Properties of Alkali Activated Low-Carbon Green Concrete

**DOI:** 10.3390/ma15175984

**Published:** 2022-08-30

**Authors:** Shaoyun Xu, Peiwei Gao, Lingling Huang, Longlong Tang, Xingqing Gu, Limin Wang

**Affiliations:** 1 Department of Civil and Airport Engineering, College of Civil Aviation, Nanjing University of Aeronautics and Astronautics, Nanjing 211106, China; 2 College of Architectural Engineering, Yangzhou Polytechnic Institute, Yangzhou 225127, China

**Keywords:** alkali excitation, green concrete, compressive strength, shrinkage properties, microstructure

## Abstract

This paper describes orthogonal experiments to investigat the effects of content of fly ash and slag, sol ratio, modulus of sodium silicate and expander on the compressive strength and shrinkage of alkali activated low-carbon green concrete (AAGC) of different ages. The microstructures and hydration product compositions of AAGC with different proportions were further studied by Scanning Electron Microscopy (SEM), X-ray Diffraction (XRD) and Mercury Intrusion Porosimetry (MIP). The results show that with an increase of fly ash content, the compressive strength of AAGC gradually decreases, the decline of compressive strength at 28 d is smaller than that of 7 d, and the shrinkage strain gradually increases at 28 d. As the sol ratio increases, the compressive strength increases first and then decreases. When the sol ratio is 0.42, the compressive strength is maximal at 28 d; the same is true for compressive strength at 7 d. Additionally, an increase of sol ratio can reduce the shrinkage strain at 28 d. Finally, when the sol ratio was 0.46, the shrinkage decreased by 30.5% compared with 0.40 at 28 d. As the modulus of sodium silicate (M_s_) increases, the compressive strength first increases and then decreases. When M_s_ is 1.4, the compressive strength reaches the maximum. As M_s_ increases, the shrinkage strain decreases first and then increases at 28 d. When M_s_ is 1.0, the shrinkage strain is the maximum at 28 d. Finally, with an increase in the content of expander, the compressive strength decreases at 7 d and 28 d, and the shrinkage strain decreases at 28 d. The shrinkage strain at 28 d is the minimum with 9% content. AAGC mixed with a small amount of fly ash and expander has more hydration products and significantly reduced cracks. In addition, the proportion of small hole volume of AAGC increases, while the proportion of large hole volume decreases. AAGC mixed with fly ash and slag without expander has more unhydrated particles and its structure is loose.

## 1. Introduction

Concrete is the most widely used building material, but a lack of resources and the environmental pollution it causes make the shortcomings of Portland cement more obvious. Compared with ordinary cement, alkali-activated, low-carbon green material, as a new cementitious material, has excellent performance, and its production is more energy efficient. According to the statistics of CO_2_ emissions, the amount of CO_2_ generated by alkali-excited cementitious materials and silicates in the production process is only about 1/5 that of Portland cement [1,2]. The quantities of industrial solid waste materials commonly used as alkali activated materials are increasing year by year. Therefore, accelerating the utilization of industrial solid waste is an inevitable requirement for sustainable development, and alkali-activated cementitious materials have been proved to have equivalent or even better performance than Portland cement [3,4,5]. Due to their low-carbon, environmentally friendly, and low-cost production process, they are considered in the material industry all over the world as one of the hottest new green gel materials of the 21st century. In addition, they have a wide range of application prospects [6,7,8].

Scholars around the world have conducted a great deal of experimental research on the performance of alkali activated cementitious material concrete. It has been found that the amount of fly ash [9], slag [10], alkali activator [11], sol ratio [12] and admixture [13] all have a great influence on the shrinkage of alkali activated concrete. The shrinkage of alkali activated fly ash and slag concrete with a sol ratio of 0.42 is smaller than that with a sol ratio of 0.34, that is, with an increase of sol ratio, the drying shrinkage decreases [14]. However, the drying shrinkage of alkali activated slag cementitious material is much larger than that of ordinary cement [15,16]. The modulus of sodium silicate decreases, as do those of alkali activated fly ash and fly ash plus slag concrete [17,18]. Some scholars have found that when 30% limestone is added to alkali activated slag concrete, shrinkage increases, but when the limestone content is increased to 50%, the shrinkage decreases [19]. The drying shrinkage of alkali excited fly ash plus slag concrete mainly occurs in the first 7 d before subsequently slowing down, reaching 1045 με after half a year; ultimately, the total shrinkage is less than that of ordinary concrete [20]. Alkali excited slag concrete undergoes significant shrinkage in the first 7 d; the reason for this is that the internal relative humidity decreases rapidly, which causes a rapid decrease in the surface tension [21]. The use of an expander is effective for dry shrinkage compensation with alkali slag concrete; in this way the dry shrinkage rate can be reduced proportionally to the amount of expander added. However, the addition of an expansion agent also slightly reduces the strength of the concrete [22]. Composite systems of alkali activated fly ash and slag have better performance than any single system [23,24]. When the slag content is more than 0.7, the setting time decreases significantly, while the shrinkage cracks on the surface of the specimen increase [25,26]. Research has shown that increasing the content of fly ash in fly ash slag mixed cementitious material will prolong the setting time, reduce the drying shrinkage and compressive strength, and improve the processing performance. Fly ash can increase the volume stability of cementitious material because spherical fly ash particles serve as a micro aggregate [27].

Previous research on the durability of alkali activated concrete has focused on acid resistance, impermeability, freeze-thaw, carbonation and other aspects [28,29,30,31,32]. As such, there is a lack of research on its shrinkage properties. The cracking caused by shrinkage will affect the life of the component. In order to correctly evaluate the early cracking risk of alkali excited green concrete, it is necessary to study its shrinkage performance. Such research will also promote the maturity of alkali excited green cementitious material technology to a certain extent.

To date, few studies have examined the effects of the composite proportion of fly ash and slag, sol ratio, modulus of sodium silicate and expander on alkali activated low-carbon green concrete. In this paper, alkali activated low-carbon green concrete was prepared by orthogonal experiments. Such experiments were designed to evaluate the effects of the composite ratio of fly ash and slag, sol ratio, modulus of sodium silicate and expander on the compressive strength and shrinkage properties of alkali activated low-carbon green concrete of different ages. In addition, the microstructures and product compositions of alkali activated low-carbon green concrete with different proportions were further studied by scanning electron microscopy (SEM), X-ray diffraction (XRD) and mercury intrusion porosimetry (MIP).

## 2. Experiment Materials and Methods

### 2.1. Materials

The performance indicators of the slag and fly ash, produced by Nanjing company, met the requirements of the GB/T18046-2000 and GB/T1596-2005 Chinese standards, respectively. The chemical composition, as provided by the manufacturer, is shown in Table 1. Sodium silicate, with a modulus (M_s_) of 3.24 and Na_2_O and SiO_2_ contents of 8.73 wt% and 27.45 wt%, respectively, was produced by a company in Shanghai. M_s_ was adjusted by adding NaOH. The specific indicators are shown in Table 2. Analytically pure NaOH, i.e., the main component content was greater than 99%, was produced by a company in Shanghai. River sand with fineness modulus of 2.58 and basalt gravel with particle size of 5–20 mm were used. The expander was produced by a company in Nanjing; the performance indicators are shown in Table 3. The expander met the requirements of GB50119-2013.

### 2.2. Specimen Preparation and Testing

In order to research the effect of fly ash content, sol ratio, Ms and expander content on the performance of AAGC, the L_16_ (4^4^) orthogonal test was adopted. Table 4 and Table 5 list the orthogonal test factor levels and AAGC mix ratios, respectively.

Demolded cubic specimens of 100 mm × 100 mm × 100 mm were placed in a curing chamber for 7 d and 28 d, respectively. The temperature was maintained at 20 °C and the relative humidity was above 95%. The specimens were used to measure compressive strength, according to the relevant test methods in GB-T50081-2011, with a 500 t micro-electro-hydraulic servo universal testing machine.

The shrinkage deformation at different ages was tested according to GB/T 50082-2009. The size of the specimen was 55 × 55 × 280 mm^3^, with a probe installed at both ends. The specimen was demolded after forming for 24 h ± 2 h. Then, it was placed in a curing box to allow continued curing for 3 d at 20 °C and RH ≥ 95%, Next, its initial length (*L*_0_) was measured using a micrometer. Subsequently, curing continued and the length (*L_t_*) of the specimen was measured at different ages. The shrinkage strain was calculated using Equation (1):

(1) $$\varepsilon_{st}=\frac{L_{\;0}-{\;L}_{t}}{L}$$

where *ε_st_* is shrinkage strain of specimens at different ages, *L* is the measuring distance of the specimen, i.e., the sum of the length of the concrete specimen minus the embedding depth of the two probes, in mm.

The 5 mm × 5 mm × 5 mm samples that had been cured for 28 d were immersed in absolute ethanol for 3 d before being taken out and dried in a vacuum at 60 °C for 48 h. A JSM-6510 high-resolution scanning electron microscope was used for SEM scanning analysis.

Samples were taken from a crushed rectangular specimen that had been cured for 28 d, immersed in absolute ethanol for 3 d, dried in a vacuum at 60 °C for 24 h, partially ground to powder and sieved through an 80-μm sieve. An XRD test was then used to analyze the phases of the specimen under a scanning range of 10° to 80° at a scanning rate of 0.30 s/step using a Bruker-Axs D8 DISCOVER with step size = 0.02°. The samples were analyzed by mercury intrusion, performed using an Auto Pore IV 9510 mercury injection meter produced by Micromeritics Instrument Corporation (Norcross, GA, USA).

## 3. Results and Discussion

### 3.1. Compressive Strength

**Figure 1:**
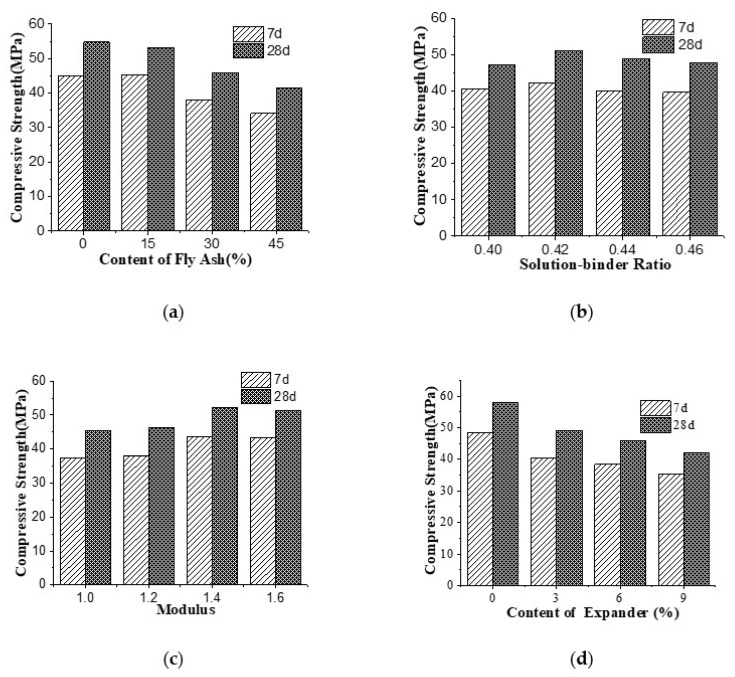
The effect of different factors on the compressive strength of AAGC: (**a**) Fly ash (**b**) Sol ratio (**c**) Modulus and (**d**) Expander.

The effects of fly ash content, sol ratio, M_s_ and expander content on the compressive strength of AAGC at 7 d and 28 d are shown in Figure 1.

Figure 1a shows that as the content of fly ash increased, the compressive strength of AAGC decreased gradually. As the content of fly ash increased from 0 to 45%, the compressive strength of samples cured for 7 d and 28 d decreased from 48.3 to 35.4 MPa and from 58.0 to 42.1 MPa, respectively. The main reason for this is that slag has more active oxides than fly ash, and the CaO content of fly ash is low. With the increase of fly ash content, the activity of green cementitious materials decreased, as did the hydration rate and quantity of hydration products. Additionally, the calcium–silicon ratio in the C-S-H gel decreased after hydration. It can be seen from the figure that the strength development of AAGC slowed due to the addition of fly ash, and that the decline in compressive strength at 28 d was smaller than that of 7 d.

As illustrated in Figure 1b, with the increase of sol ratio, the compressive strength showed an increasing and then a decreasing trend at 7 d and 28 d. When the sol ratio was 0.42, the compressive strength reached the maximum at 28 d; the same was true for compressive strength at 7 d.

As shown in Figure 1c, with the increase in M_s_ from 1.0 to 1.4, the compressive strength of AAGC cured for 7 d and 28 d increased. When sodium silicate and slag are mixed, the former hydrolyzes to form NaOH and Si(OH)_4_. The Si(OH)_4_ silica gel is then transformed into active SiO_2_ and H_2_O, so the amount of active SiO_2_ is determined by M_s_. OH^−^ penetrates the surface of slag in alkaline solution, enters the vitreous body, dissociates the vitreous body and dissociates Ca^2+^ and SiO_4_^4−^, because Ca^2+^ has a high diffusion speed and reacts with SiO_4_^4−^ to form C-S-H gel. The M_s_ directly affects the amount of SiO_2_ and active OH^−^, and the activation effect is determined by SiO_2_ and active OH^−^. Therefore, when the M_s_ increases, the compressive strength increases. However, when the M_s_ is too great, the calcium–silicon ratio decreases, as do the quantity of hydration products and the compressive strength of the concrete. In summary, when the M_s_ reaches 1.6, the balance of the two-sided effect of silica on the compressive strength is broken and the compressive strength decreases.

Figure 1d shows that as the content of expander was increased, the compressive strength of AAGC cured for 7 d and 28 d decreased gradually. As the content of expander increased from 0 to 9%, the compressive strength of the samples cured for 7 d and 28 d decreased from 44.9 to 34.2 MPa and from 54.8 to 41.3 MPa, respectively. The reason for this is that the formed specimen is unconstrained, the hydration speed of calcium oxide is slow; after alkali excitation cementitious materials coagulate and harden, calcium oxide particles then continue to hydrate, and the hydration products damage the already coagulated and hardened structure. In addition, with the increase of the content of expander, CaO forms Ca(OH)_2_, which increases the expansion of concrete, thus destroying the interface structure and reducing the compressive strength.

### 3.2. Drying Shrinkage

**Figure 2:**
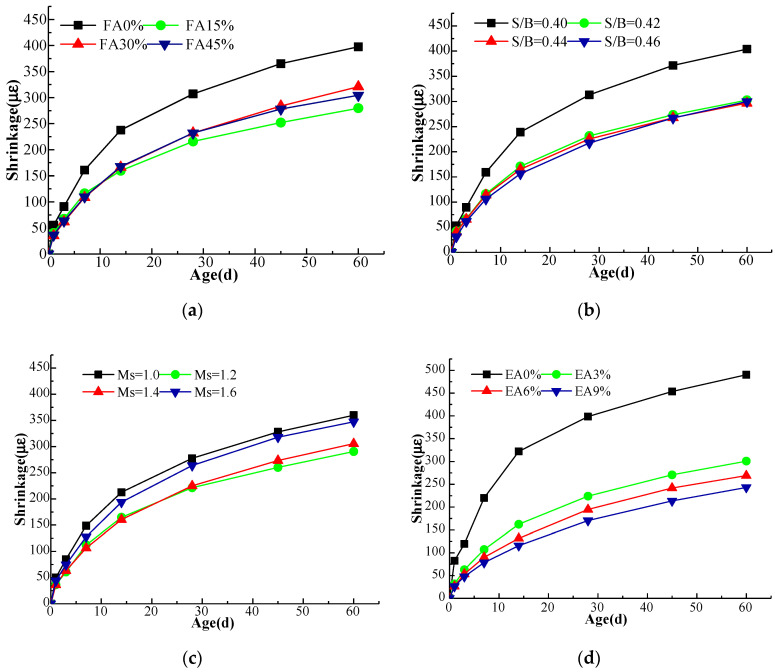
Influence of shrinkage strain under standard curing (**a**) Fly ash (**b**) Sol ratio (**c**) Modulus (**d**) Expander.

The effects of fly ash content, sol ratio, M_s_ and expander content on the drying shrinkage of AAGC are displayed in Figure 2.

Figure 2a shows that AAGC without fly ash underwent the most shrinkage. When the fly ash content was 15%, 30% and 45%, respectively, the shrinkage comprised 70.3%, 75.6% and 75.6% of the volume of the concrete with 15% fly ash content at 28 d. This shows that the addition of fly ash can significantly inhibit the shrinkage of AAGC. The main reason for this is that fly ash has low activity, thereby inhibiting the hydration rate and improving the pore structure. The hydration of fly ash requires relatively less water, and the particle gradation formed by the mixing of fly ash and slag is better, which also reduces the area of contact with water and is conducive to inhibiting the shrinkage deformation of a fly ash and slag composite system. At the same time, the density of the C-S-H gel in AAGC mixed with fly ash was lower than that of the pure slag material. Under the same conditions, the volume of C-S-H gel generated in the composite system was larger than that of pure slag, which played a role in compensating for the shrinkage.

As illustrated in Figure 2b, when the sol ratio was 0.42, 0.44 and 0.46, shrinkage was reduced by 26.0%, 28.0% and 30.5% compared with a 0.40 sol ratio at 28 d, respectively. This shows that the shrinkage deformation of AAGC can be effectively reduced by adjusting the sol ratio.

As shown in Figure 2c, when the M_s_ increased from 1.0 to 1.2, 1.4 and 1.6, the shrinkage strain of AAGC decreased by 20.3%, 19.0% and 4.9% at 28 d, respectively. When the M_s_ was 1.2, the shrinkage strain reached the minimum at 28 d. The M_s_ has a dual effect on the shrinkage deformation of AAGC. On the one hand, an increase of M_s_ can increase the porosity; as such, the chances of internal water movement are increased, and therefore, shrinkage decreases due to the presence of sufficient moisture in the specimen. On the other hand, the increase of M_s_ can accelerate the hydration rate of alkali activated materials and increase shrinkage. Therefore, the shrinkage strain decreased and then increased with an increase of M_s_ at 28 d.

Figure 2d shows that as the content of expander increased, the shrinkage strain decreased gradually at 28 d. When the content of expander was 3%, 6% and 9%, the shrinkage strain accounted for 56.2%, 48.8% and 42.7%, respectively, of that of the specimen without expander at 28 d. The addition of expander promotes the formation of new substances to produce volume expansion which compensates for shrinkage deformation, improves the pore structure of AAGC, refines the size of the pores, and thus, reduces shrinkage.

### 3.3. Microscopic Analysis of Alkali-Activated Green Concrete

#### 3.3.1. SEM Analysis

**Figure 3:**
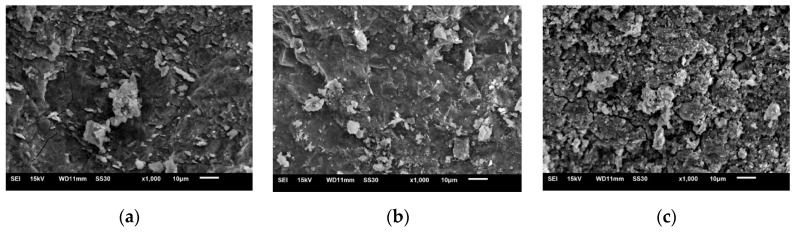
SEM image of specimens after standard curing for 28 d. (**a**) G1. (**b**) G8. (**c**) G12.

The G1, G8 and G12 samples, cured for 28 d, were selected for SEM analysis. As shown in Figure 3, after the hydration of the cementitious material, AAGC produced a large number of hydration products, including needle-like and massive, clustered particles comprising hydration products. However, unhydrated substances could still be seen under high-power SEM, and a small number of microcracks with different widths and lengths were observed.

In G1, i.e., pure slag without expander, a small number of micro cracks could be seen. In G8, i.e., mixed with a small amount of fly ash and expander, the structure was more dense, the number of cracks was significantly reduced, and there were more hydration products. In G12, i.e., with more fly ash and without expander, many additives were not hydrated. Additionally, at a magnification of 1000 times, wide cracks could clearly be seen, and the particles did not form a whole, as hydrated gel products and unreacted particles cannot bond tightly together, giving rise to a loose structure.

#### 3.3.2. XRD Analysis

**Figure 4:**
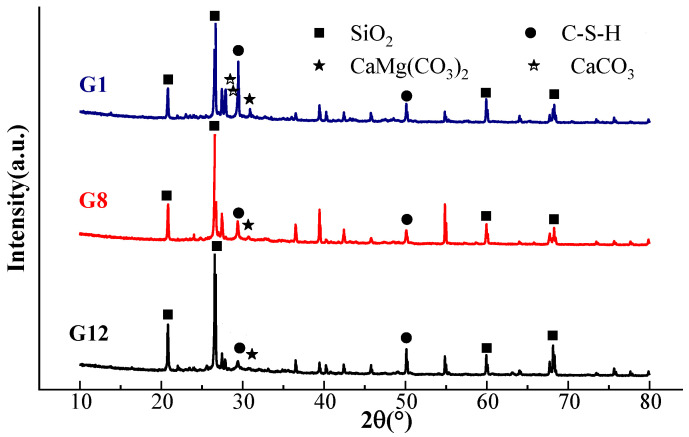
XRD spectrum after standard curing for 28 d.

Figure 4 shows the XRD patterns of G1, G8 and G12. It can be seen that the three groups of AAGC had characteristic diffraction peaks of calcium silicate hydrate (C-S-H), indicating that the main product of AAGC hydration was a C-S-H gel. A little calcite (CaCO_3_) was found in G1, indicating that the specimen had undergone a slight carbonation reaction during the molding hydration process. The reason for this was that the content of CaO in pure slag alkali activated cementitious materials was high, which led to the carbonation of the generated Ca(OH)_2_. The diffraction peak intensity of G8 was weaker than that of G1 because of the addition of fly ash. The hydration diffraction peak of G12 was significantly weaker than those of G1 and G8. It can be seen that with an increase of the content of fly ash, crystallization of hydration products gradually decreased. The reason for this is that the activity of fly ash is lower than that of slag, and its hydration rate is lower. Over time, the strength of AAGC could be improved by further hydration at a later stage. The main hydration product of AAGC is C-S-H gel with small amounts of Ca/Si. There is a small amount of C-A-S-H gel with low Ca/(Si + Al) in fly ash and slag systems.

#### 3.3.3. MIP Analysis

**Figure 5:**
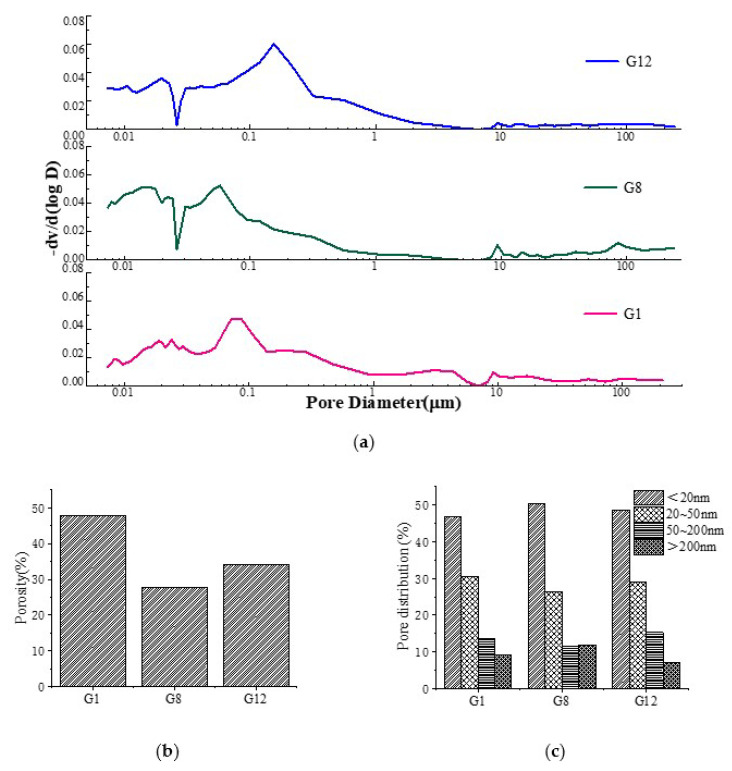
Pore size distribution curve after 28 d of standard curing: (**a**) Pore size distribution curve; (**b**) Porosity; (**c**) Pore proportion.

The pore size distributions of G1, G8 and G12 are shown in Figure 5. The volume content of pores smaller than 50 nm in the three groups of samples was significantly more than that of pores larger than 50 nm. Compared with G1 and G8, it can be seen that the addition of expander reduced the porosity of AAGC and improved the pore size distribution and pore structure characteristics. This was due to the hydration products in the expander filling the internal pores, thereby reducing the porosity and improving the aperture distribution.

## 4. Conclusions

Based on experimental research and a theoretical analysis, the following conclusions were obtained:

(1) With an increase of fly ash content, the compressive strength of AAGC gradually decreased. The decline in compressive strength at 28 d was smaller than that at 7 d. As the content of fly ash increased from 0 to 45%, the compressive strength of the AAGC at 7 d and 28 d decreased from 48.3 to 35.3 MPa and from 58.0 to 42.1 MPa, respectively. With an increase of sol ratio, the compressive strength increased and then decreased at both 7 d and 28 d. When the sol ratio was 0.42, the compressive strength reached the maximum at 28 d; the same was true for compressive strength at 7 d. When the M_s_ increased, the compressive strength first increased and then decreased at 7 d and 28 d. When the M_s_ was 1.4, the compressive strength reached the maximum at 28 d. Finally, the compressive strength of AAGC decreased with an increase of expander content at 7 d and 28 d.
(2) As the content of fly ash increased, the shrinkage strain of AAGC increased gradually at 28 d. The shrinkage strain showed no obvious change when the content was more than 30%. The shrinkage strain could be reduced with an increase of sol ratio at 28 d. When the sol ratio was 0.46, the shrinkage strain reached the minimum at 28 d. With an increase of M_s_, the shrinkage strain of AAGC first decreased and then increased at 28 d. When M_s_ was 1.2, the shrinkage strain reached the minimum at 28 d. Finally, as the content of expander increased, the shrinkage strain decreased gradually at 28 d.
(3) When a small amount of fly ash and expander were added to AAGC, there were more hydration products, and the extent internal micro-cracks was significantly reduced. The AAGC only mixed with fly ash contained many unhydrated particles, and the structure was loose. The main hydration product of AAGC was found to be C-S-H gel. There were some carbonation reactions in AAGC without fly ash, and crystallization decreased with an increase of fly ash content. The addition of fly ash greatly reduced the porosity, as did the addition of expander. In addition, the proportion of small hole volume increased, while that of large hole volume decreased.

## Figures and Tables

**Table 1:** Chemical composition of slag and fly ash (%).

Materials	CaO	SiO_2_	Al_2_O_3_	MgO	SO_3_	TiO_2_	Fe_2_O_3_	MnO	Others
Slag	43.26	29.32	16.78	7.04	1.71	0.88	0.55	0.46	-
Fly ash	5.65	53.21	32.36	1.58	2.23	-	2.87	-	2.1

**Table 2:** Properties of water glass.

Target	Modulus	Baume Degree	Density	SiO_2_ (%)	Na_2_O (%)	Insoluble (%)	Iron Content (%)
Value	3.1–3.4	39.2–40.2	1.368–1.394	≥26	≥8.2	≤0.2	≤0.02

**Table 3:** Properties and composition of calcium oxide expansion agent.

Component (%)					Setting Time (min)	
CaO	Sieving Residue	Humidity	Alkali Content	Cl^−^	Initial Set	Iinal Set
≥98	≤0.50	≤0.40	≤0.20	≤0.03	165	350

**Table 4:** Orthogonal test factor levels.

NO.	Fly Ash Content (FA%)	Sol Ratio (S/C)	Modulus (M_s_)	Expander (EA%)
1	0	0.40	1.0	0
2	15	0.42	1.2	3
3	30	0.44	1.4	6
4	45	0.46	1.6	9

**Table 5:** AAGC mix ratios (kg/m^3^).

NO.	Fly Ash (kg/m^3^)	Slag (kg/m^3^)	Alkali Solution (kg/m^3^)	Modulus (Ms)	Expander (kg/m^3^)	Sand (kg/m^3^)	Gravel (kg/m^3^)
G1	0	400	160	1.0	0	716	1074
G2	0	400	168	1.2	12	716	1074
G3	0	400	176	1.4	24	716	1074
G4	0	400	184	1.6	36	716	1074
G5	60	340	160	1.4	24	716	1074
G6	60	340	168	1.6	36	716	1074
G7	60	340	176	1.0	0	716	1074
G8	60	340	184	1.2	12	716	1074
G9	120	280	160	1.6	36	716	1074
G10	120	280	168	1.4	24	716	1074
G11	120	280	176	1.2	12	716	1074
G12	120	280	184	1.0	0	716	1074
G13	180	220	160	1.2	12	716	1074
G14	180	220	168	1.0	0	716	1074
G15	180	220	176	1.6	36	716	1074
G16	180	220	184	1.4	24	716	1074
